# *Bordetella trematum* infection: case report and review of previous cases

**DOI:** 10.1186/s12879-019-4046-8

**Published:** 2019-05-30

**Authors:** Thaís Regina y Castro, Roberta Cristina Ruedas Martins, Nara Lúcia Frasson Dal Forno, Luciana Santana, Flávia Rossi, Alexandre Vargas Schwarzbold, Silvia Figueiredo Costa, Priscila de Arruda Trindade

**Affiliations:** 1 Universidade Federal de Santa Maria, Santa Maria, Brazil; 2 Faculdade de Medicina da Universidade de São Paulo, São Paulo, Brazil; 3 Hospital Universitário de Santa Maria, Santa Maria, Brazil; 4 Hospital das Clínicas da Faculdade de Medicina da Universidade de São Paulo, São Paulo, Brazil; 5 Departamento de Análises Clínicas e Toxicológicas, Centro de Ciências da Saúde, Universidade Federal de Santa Maria, Avenida Roraima, 1000, Camobi, Santa Maria, RS CEP 97105-900 Brazil

**Keywords:** Foot ulcer, diabetic, *Bordetella* infection, Antimicrobial, Susceptibility breakpoint determination

## Abstract

**Background:**

*Bordetella trematum* is an infrequent Gram-negative coccobacillus, with a reservoir, pathogenesis, a life cycle and a virulence level which has been poorly elucidated and understood. Related information is scarce due to the low frequency of isolates, so it is important to add data to the literature about this microorganism.

**Case presentation:**

We report a case of a 74-year-old female, who was referred to the hospital, presenting with ulcer and necrosis in both legs. Therapy with piperacillin-tazobactam was started and peripheral artery revascularization was performed. During the surgery, a tissue fragment was collected, where *Bordetella trematum*, *Stenotrophomonas maltophilia,* and *Enterococcus faecalis* were isolated. After surgery, the intubated patient was transferred to the intensive care unit (ICU), using vasoactive drugs through a central venous catheter. Piperacillin-tazobactam was replaced by meropenem, with vancomycin prescribed for 14 days. Four days later, levofloxacin was added for 24 days, aiming at the isolation of *S. maltophilia* from the ulcer tissue. The necrotic ulcers evolved without further complications, and the patient’s clinical condition improved, leading to temporary withdrawal of vasoactive drugs and extubation. Ultimately, however, the patient’s general condition worsened, and she died 58 days after hospital admission.

**Conclusions:**

Despite being a rare finding, *B. trematum* is typically associated with the clinical manifestation of disorders that predispose to ulcer development, which can be infected by microorganisms. The combination of antibiotic therapy and surgical debridement plays a key role in preventing systemic infections. Monitoring the appearance of new cases of *B. trematum* is essential, since it can be an emerging microorganism. Isolating and defining the clinical relevance of unusual bacteria yields a more accurate perspective in the development of new diagnostic tools and allows for assessment of proper antimicrobial therapy.

## Background

*Bordetella trematum* is an infrequent gram-negative coccobacillus [1], typically related to tissue infections. Related information is scarce due to the low frequency of isolates [2]. As laboratories gain greater access to technologies for accurate and specific bacterial identification, rare microorganisms can arise. It is essential to understand the clinical significance of these unusual findings and the need for treatment. We reviewed the published case reports and will be discussing a new one here.

## Case presentation

A 74-year-old female patient attended the vascular surgery outpatient clinic and was referred to the hospital for revascularization of the distal arteries. She had necrotic ulcers in both legs, worse in the right. She reported pain, signs of local infection and myiasis on the lateral side of the ankle, tendon exposure, edema, and dry skin, but no signs of acute ischemia. Her underlying diseases were difficult to control: systemic arterial hypertension for 20 years; type II diabetes mellitus (DM) for 13 years; hypothyroidism; a stroke 6 years ago, chronic renal failure class IV; peripheral arterial occlusive disease, and postmenopausal osteoporosis. The patient referred to previous angioplasty performed 1 year earlier on the lower right leg due to peripheral arterial occlusive disease. Upon hospital admission, several sites of infection other than skin and soft tissue were discarded. Laboratory tests showed a normal leukocyte count and reactive C protein of 3.98 mg/dL (reference value: < 0.30 mg/dL). Empiric treatment with piperacillin-tazobactam (4.5 g IV 6/6 h) was initiated, which was prescribed for 5 days.

Two days after admission, surgical debridement was performed. Limb amputation was discussed, but rejected by the patient and family members. During the surgery, a fragment of the ulcer tissue was collected and sent to the hospital’s microbiology laboratory. In the staining procedure, a few gram-positive cocci and gram-negative bacilli were observed. The specimen was submitted for enrichment in the brain-heart infusion broth for 24 h/37 °C and later seeded in 5% sheep blood agar and MacConkey agar, incubated for 37 °C, and presented growth after 24 h. VITEK 2 system (bioMérieux, Marcy l’Etoile, France) identified *Enterococcus faecalis, Stenotrophomonas maltophilia,* and *B. trematum*. The isolate was subsequently identified as *B. trematum,* using VITEK MS (bioMérieux, Marcy l’Etoile, France) and confirmed by 16S rRNA gene sequencing with Illumina MiSeq (Illumina_,_ San Diego, CA, USA). The oxidase test was negative. MICs were determined by Sensititre gram-negative MIC plate (Thermo Scientific, Waltham, MA, USA) (Table 1).

**Table 1:** Case reports associated with *Bordetella trematum*

Case report	Clinical information	Infection isolate(s)	Susceptibilty profile of *B. trematum*^a,b^	Therapy^a^	Outcome
Daxboeck et al. (2004) [3]	Male, 82-year-old, with type 2 diabetes mellitus (DM), and infected ulcer on left foot.	*Bordetella trematum* and *Pseudomonas aeruginosa*	Susceptible to AMI, AMC, CTX, CAZ, GEN, IMI, TZP. Resistant to CXM and CIP.	10-day treatment of AMC and CIP; without clinical improvement; the antimicrobial treatment was discontinued.	Favorable
Hernández-Porto et al. (2013) [2]	Female, 76-year-old, with DM, renal failure, peripheral vascular disease, and ulcers in lower extremities	*B. trematum* and *Achromobacter xylosoxidans*	Susceptible to AMI (16 μg/mL), AMC (2 μg/mL), CAZ (8 μg/mL), GEN (4 μg/mL), IMI (0,5 μg/mL), MEM (0.125 μg/mL), TZP (1 μg/mL) and SXT (0.5 μg/mL) Resistant to ATM (> 32 μg/mL), CTX (> 32 μg/mL), CXM (4 μg/mL) and CIP (4 μg/mL).	21 days with SXT and 14 days with CAZ (initiated 2 weeks after positive culture for *B. trematum*).	Favorable
Halim et al. (2014) [4]	Male, 60-year-old, no history of pathologies, presenting thorax burns by butane gas	*B. trematum* and *Enterobacter cloacae* in blood culture	Susceptible to CIP, CLA, CL, DOX, IMI, MIN and NET. Resistant to AMI, AMC, CTX, CAZ, CF, GEN, and TOB.	IMI, NET, and CL.	Death
Almagro-Molto, Eder, Schubert (Case 1) (2015) [5]	Male, 65-year-old, with DM, peripheral vascular disease and foot ulcer	*B. trematum, Staphylococcus aureus, Proteus vulgaris, Alcaligenes faecalis* and *Morganella morganii*	Susceptible to AMI, AMC, AMP, CPM, GEN, IMI, LVX, MEM, MIN, PIP, TZP, SXT, TGC, and TOB. Resistant to CTX, FOX, CAZ, CXM, CIP, ETP, and FOS.	Debridement and 7-day course of CIP	Persistence of infection
Almagro-Molto, Eder, Schubert (Case 2) (2015) [5]	Female, 72-year-old, suspected of osteomyelitis, bone defects in the feet and ankles, venous disorder, impaired renal function, ulcer in both feet	*B. trematum, P. vulgaris* and *A. faecalis* in both feet ulcer exsudate samples/*B. trematum,* MRSA, *A. faecalis* and *S. maltophilia* in the surgical sample of ulcer of both feet	Susceptible to AMI, AMC, AMP, CPM, GEN^c^, IMI, LVX^c^, MEM, MIN, PIP, TZP, SXT, TGC, and TOB. Intermediate to ETP and LVX^c^ Resistant to CTX, FOX, CAZ, CXM, CIP, FOS, and GEN^c.^	Compression therapy and 14-day course of CIP; despite clinical improvement, both limbs were amputated after 3 weeks; antimicrobial therapy with TZP (7 days) followed by MEM (7 days)	Favorable
Saksena, Manchanda, Mittal (2015) [6]	Young girl, 7-month-old, febrile, presenting vomiting; provisional diagnosis of infantile tremor syndrome with protein energy malnutrition and developmental delay	*B. trematum* in blood culture	Susceptible to AMS (8 μg/mL), CIP (1 μg/mL), IMI (1 μg/mL), TZP (8. μg/mL) and SXT (20 μg/mL) Intermediate to CPM (16 μg/mL), CRO (32 μg/mL), LVX (4 μg/mL), PIP (64 μg/mL) and TOB (8 μg/mL) Resistant to AMI (64 μg/mL), CAZ (64 μg/mL), CL (16 μg/mL), GEN (16 μg/mL), MEM (16 μg/mL)	Empirical therapy with CRO for 5 days; then the treatment switched to TZP and AMI; on the 12th day, therapy was modified to CIP and AZM (5 days)	Favorable
Almuzara et al. (2015) [7]	Male, 14-year-old, febrile and hemodynamically unstable, presenting left hip septic arthritis. Diagnosis of chronic osteomyelitis (*S. aureus*)	*Escherichia coli* and *B. trematum* in bone biopsy	Susceptible to AMI (16 μg/mL), CPM (4 μg/mL), CAZ (4 μg/mL), CF (8 μg/mL), CL (≤ 5 μg/mL), GEN (4 μg/mL), IMI (≤ 1 μg/mL), MEM (≤ 0.25 μg/mL) and SXT (≤ 2 μg/mL) Intermediate to AMP (16 μg/mL), AMS (16 μg/mL), CTX (32 μg/mL) and CIP (2 μg/mL)	Multiple surgical debridement, antimicrobial treatment with MEM and SXT for 6 months; MIN later	Favorable
Majewski et al. (2016) [8]	Transgender male, 61-year-old, with below-knee amputations in both lower limbs, DM, stage IV chronic kidney disease, and coronary artery disease, with a right femur fracture after a fall, respiratory distress, septic shock and worsening of left leg wound after hospitalization	*B. trematum* in blood culture	Susceptible to AMI, AMC, CAZ (8 μg/mL), CIP (0,008 μg/mL), IMI (0.25 μg/mL), LVX (0,03 μg/mL), TZP (2 μg/mL) and TOB Intermediate to CTX (16 μg/mL) Resistant to CXM and GEN	Empirical treatment with TZP and VAN; the left lower limb infection worsened, treatment switch to CIP; due to necrotizing fasciitis probability CLI was added; persistence of septic shock, TOB added	Death
Current report	Female, 74-year-old, with necrotic ulcers in both legs, presenting signs of local infection, systemic arterial hypertension; DM; hypothyroidism; peripheral arterial occlusive disease; stage IV chronic kidney disease	*B. trematum, E. faecalis* and *S. maltophilia* in a tissue fragment	MIC ≤025 μg/mL to TGC, CLI and PB; MIC 0.5 μg/mL to DOR; MIC 1 μg/mL to CIP, LVX, IMI and MEM; MIC 2 μg/mL to DOX, GEN, MIN, TOB and CPM; MIC 4 μg/mL to AMI and CAZ; MIC 8 μg/mL to CTX; MIC 4/2 μg/mL to AMS; MIC 8/4 μg/mL to TZP; MIC 9.5/4.5 μg/mL to SXT MIC 16/2 μg/mL to TIM; MIC > 16 μg/mL to ATM	Empirical treatment with TZP and surgical debridement; treatment switch to VAN and MEM due to septic shock; LVX added aiming *S. maltophilia*	Death

^a^*AMI* amikacin, *AMC* amoxicillin-clavulanic acid, *AMP* ampicillin, *AMS* ampicillin-sulbactam, *AZM* azithromycin, *ATM* aztreonam, *CPM* cefepime, *CTX* cefotaxime, *FOX* cefoxitin, *CAZ* ceftazidime, *CRO* ceftriaxone, *CXM* cefuroxime, *CIP* ciprofloxacin, *CLA* clarithromycin, *CLI* clindamycin, *CF* cephalothin, *CL* colistin, *DOR* doripenem, *DOX* doxycycline, *ETP* ertapenem, *FOS* fosfomycin, *GEN* gentamicin, *IMI* imipenem, *LVX* levofloxacin, *MEM* meropenem, *MIN* minocycline, *NET* netilmicin, *PB* polymyxin B, *PIP* piperacillin, *TIM* ticarcillin-clavulanate, *TZP* piperacillin-tazobactam, *SXT* sulfamethoxazole-trimethoprim, *TGC* tigecycline, *TOB* tobramycin, *VAN* vancomycin, *MIC* minimum inhibitory concentration, *MRSA* methicillin-resistant *Staphylococcus aureus*

^b^Methodology for susceptibility testing was the E-test [2, 3, 5, 6]; disk-diffusion [2, 4, 8]; broth microdilution [3, 6]; VITEK 2 [5, 7]

^c^After amputation of lower limbs, change was seen in susceptibility profile in the second isolate

After surgery, the intubated patient was transferred to the ICU, using vasoactive drugs through a central venous catheter. Three days later, she presented a worsening clinical condition. Oxacillin-resistant *Staphylococcus hominis* was isolated from a blood culture drawn through a peripheral vein. Piperacillin-tazoctam was replaced by meropenem (500 mg IV 24/24 h) and vancomycin (1 g IV 24/24 h), prescribed for 14 days. Four days later, levofloxacin (750 mg IV 24/24 h) was added for 24 days aiming at *S. maltophilia* isolated from the ulcer tissue.

**Fig. 1:**
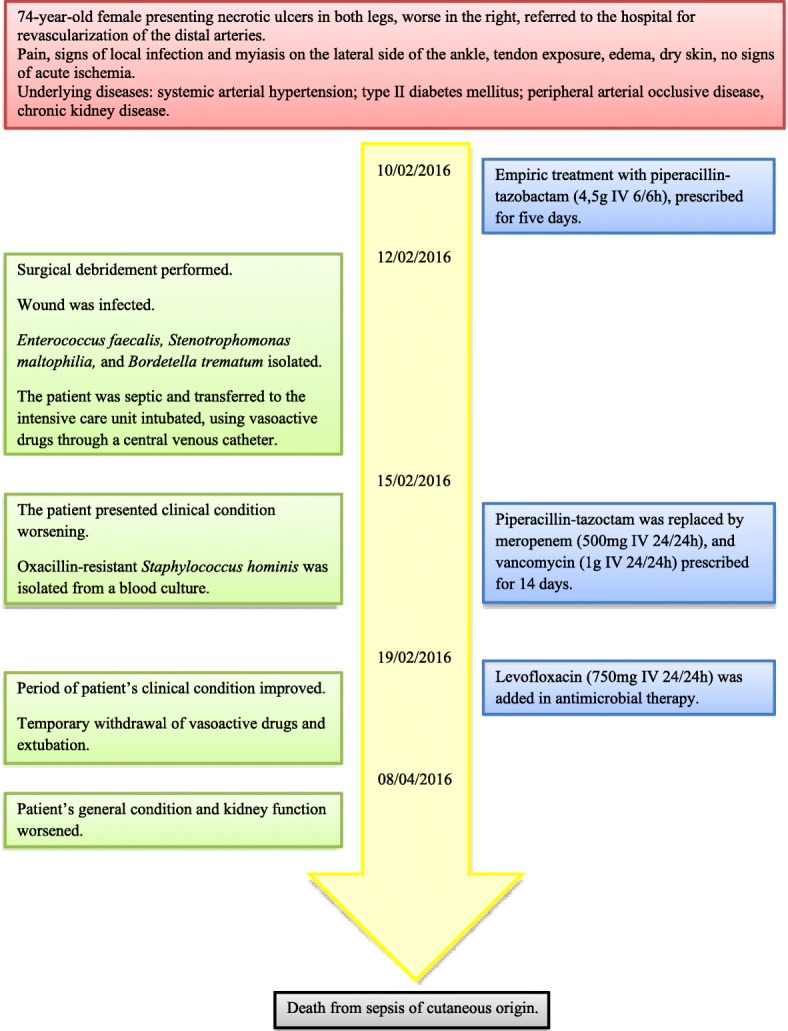
A timeline of the main events of the patient’s illness

The necrotic ulcers evolved without further complication and the patient’s clinical condition improved, leading to temporary withdrawal of the vasoactive drugs and extubation. However, the patient’s general condition and kidney function worsened, probably due to the severity of her underlying diseases, and she died from sepsis of cutaneous origin 58 days after hospital admission. Autopsy was not performed. Figure 1 shows a timeline of the events.

## Discussion and conclusions

*B. trematum* was described in 1996, isolated from human wounds and ear infections [1]. Information about its reservoir, life cycle and pathogenesis remain unknown. Regarding virulence, little is known [5]. Typically associated with tissue infections in diabetic patients, and generally occurs in polymicrobial infections, which further complicates its clinical interpretation [2]. In our case, where *E. faecalis* and *S. maltophilia* were isolated with *B. trematum* in the ulcer, the role of *B. trematum* in the patient’s prognosis became unclear. However, as this microorganism was previously reported as a causative agent of bloodstream infection [4, 6, 8], its interpretation and implication in disease was challenging and required integration of clinical, epidemiological, and microbiological issues.

When first described, *B. trematum* presented the following phenotypic characteristics: non-glucose metabolizer, grown on MacConkey agar, motile, with variable nitrate reduction, catalase and citrate positive, urease, oxidase and lysine decarboxylase negative [1]. Methodologies such as MALDI-TOF MS and VITEK 2 system were efficient in identifying *B. trematum*. However, there are reports that demonstrate problems regarding microorganism misidentification by API 20 NE (bioMérieux, Marcy l’Etoile, France), due to the absence of *B. trematum* in its identification database [2]. In another case, also using API 20 NE, *B. trematum* was misidentified as *Achromobacter denitrificans*/*Bordetella bronchiseptica*. This may have occurred because the nitrate reduction test was variable, and the oxidase test reagent was different from the one used by other researchers [7]. In some cases, the confirmation of the microorganism identification was carried out by 16S rRNA gene sequencing [3–5, 7, 8]. In our case, the isolate was correctly identified by the VITEK 2 system and MALDI-TOF MS and confirmed by 16S rRNA gene sequencing. Even with credible identification by using routine laboratory instruments, the available literature only reports a few cases, as summarized in Table 1.

There is no standardized methodology by the Clinical Laboratory Standard Institute (CLSI) or the European Committee on Antimicrobial Susceptibility Testing that performs an antimicrobial susceptibility test, specifically for *B. trematum*. Some authors have used the CLSI manual as an interbreeding source [2, 5–8], cited as the MIC interpretative standards for other *Non*-*Enterobacteriaceae* and MIC interpretative standards for *Enterobacteriaceae*, along with the use of antibiotics such as ampicillin and cephalothin.

Analyzing antimicrobial susceptibility tests performed by other authors (Table 1), *B. trematum* has always shown sensitivity to piperacillin-tazobactam, which was initially used as an empirical therapy. Indeed, for this antimicrobial, we obtained the same MIC (≤ 8/4 μg/mL) as reported by Saksena et al. (2015), which may have been a contributing factor to the favorable outcome of the surgical wound. Cefotaxime showed MIC 32 μg/mL in other studies [2, 7], as it is considered to be an intermediate resistant. The MIC in our study (8 μg/mL) was lower than that described by other authors. However, it is not possible to define whether this MIC represents susceptibility. The problems with MIC interpretation are probably due to lack of in vitro/in vivo susceptibility correlation or breakpoints [6].

Since *B. trematum* infection/colonization occurs primarily in wounds, debridement is essential for treatment, because it removes pathogens from nonviable tissues and reduces recurrence of the infection. Debridement associated with the correct use of antibiotics can lead to a favorable outcome [5]. However, there were no further cultures to determine whether *B. trematum* was actually eradicated.

The pathogenicity of this microorganism is not well-established, so it is unclear what the contribution of *B. trematum* was to the patient’s infection and outcome. Despite being a rare finding, *B. trematum* is typically associated with clinical manifestations of disorders that predispose to the development of ulcers that can become infected by microorganisms. The combination of antibiotic therapy and surgical debridement plays a key role in the cure, preventing systemic infections. Monitoring the appearance of new cases of *B. trematum* is essential, as it could be an emerging microorganism. Isolating and defining the clinical relevance of unusual bacteria facilitates an overall perspective towards the development of new diagnostic tools and allows for assessment of proper antimicrobial therapy.

## Abbreviations

CLSI: Clinical Laboratory Standards Institute
MIC: Minimal Inhibitory Concentration
